# Establishment and validation of a nomogram predicting the risk of deep vein thrombosis before total knee arthroplasty

**DOI:** 10.1186/s12959-024-00588-6

**Published:** 2024-02-16

**Authors:** Zehua Wang, Xingjia Mao, Zijian Guo, Guoyu Che, Changxin Xiang, Chuan Xiang

**Affiliations:** 1 Department of Orthopedic, The Second Hospital of Shanxi Medical University, Taiyuan, China; 2 Department of Basic Medicine Sciences, and Department of Orthopaedics of Sir Run Run Shaw Hospital, Zhejiang University School of Medicine, Hangzhou, China; 3 School of Health, Yuncheng Vocational and Technical University, Yuncheng, China; 4 College of Biomedical Engineering, Taiyuan University of Technology, Taiyuan, China

**Keywords:** Total knee arthroplasty, Preoperative deep vein thrombosis, Nomogram

## Abstract

**Purpose:**

This study aimed to analyze the independent risk factors contributing to preoperative DVT in TKA and constructed a predictive nomogram to accurately evaluate its occurrence based on these factors.

**Methods:**

The study encompassed 496 patients who underwent total knee arthroplasty at our hospital between June 2022 and June 2023. The dataset was randomly divided into a training set (*n* = 348) and a validation set (*n* = 148) in a 7:3 ratio. The least absolute shrinkage and selection operator (LASSO) and multivariate logistic regression analysis were used to screen the predictors of preoperative DVT occurrence in TKA and construct a nomogram. The performance of the predictive models was evaluated using the concordance index (C-index), calibration curves, and the receiver operating characteristic (ROC) curves. Decision curve analysis was used to analyze the clinical applicability of nomogram.

**Results:**

A total of 496 patients who underwent TKA were included in this study, of which 28 patients were examined for lower extremity DVT preoperatively. Platelet crit, Platelet distribution width, Procalcitonin, prothrombin time, and D-dimer were predictors of preoperative occurrence of lower extremity DVT in the nomograms of the TKA patients. In addition, the areas under the curve of the ROC of the training and validation sets were 0.935 (95%CI: 0.880–0.990) and 0.854 (95%CI: 0.697-1.000), and the C-indices of the two sets were 0.919 (95%CI: 0.860–0.978) and 0.900 (95%CI: 0.791–1.009). The nomogram demonstrated precise risk prediction of preoperative DVT occurrence in TKA as confirmed by the calibration curve and decision curve analysis.

**Conclusions:**

This Nomogram demonstrates great differentiation, calibration and clinical validity. By assessing individual risk, clinicians can promptly detect the onset of DVT, facilitating additional life monitoring and necessary medical interventions to prevent the progression of DVT effectively.

## Introduction

Venous thromboembolism (VTE), comprising deep vein thrombosis (DVT) and pulmonary embolism (PE), is a severe vascular condition associated with significant morbidity and risk, frequently resulting in serious clinical complications including sudden death and post-thrombotic sequelae [1–3]. Clinically, DVT predominantly manifests as a primary form of VTE, commonly developing in the lower extremities. Total knee arthroplasty (TKA), an efficacious remedy for end-stage knee conditions, substantially alleviates pain and improves knee function, thereby enhancing patients’ quality of life [4]. Nevertheless, TKA is regarded as a significant risk factor for postoperative VTE. Studies indicate that the incidence of asymptomatic DVT post-unprotected total knee arthroplasty varies from 40 to 85%, and the incidence of fatal PE without pharmacological intervention ranges between 0.87–1.99% [5, 6]. Therefore, taking reasonable and effective prophylactic measures is crucial to prevent the occurrence of DVT. Current clinical guidelines recommend several strategies to prevent DVT, including standardizing the operation to reduce the damage to the venous lining during the operation [7]; utilizing interventions like plantar vein pumps, intermittent pneumatic compression devices, and gradient pressure compression stockings post-operation to enhance lower limb blood flow, thereby reducing blood stasis [8]. Moreover, in the process of diagnosis and treatment, clinicians should choose and use anticoagulant drugs reasonably after weighing the advantages and disadvantages of thrombosis risk and bleeding risk [9]. With an increased focus on DVT prevention and the standardized application of preventive measures, the incidence of DVT after TKA shows a decreasing trend [7–9]. Literature reports suggest that through comprehensive preoperative assessment and appropriate prophylactic measures, the occurrence of DVT following TKA can be minimized to below 15% [10].

Numerous studies have concentrated on DVT prevention after TKA, and a variety of factors have been shown to be associated with the formation of DVT after TKA, including patient factors (age, body mass index, history of diabetes mellitus, etc.), surgical factors (surgical procedure, anesthesia, use of tourniquets, etc.), and perioperative management factors (pain control, early rehabilitation interventions, and venous thrombosis prophylaxis) [11]. Since the patient’s own factors exist before admission, and some studies have confirmed that such factors are an important cause of preoperative blood hypercoagulability in TKA [12–14], some scholars have speculated whether DVT is formed preoperatively, and this was verified by imaging screening. Xiong et al. [15] conducted preoperative ultrasound screening on 584 total knee arthroplasty patients, revealing DVT in 32 cases, indicating a 6.99% incidence (32/584), with 3 being proximal thrombi. This study identified preoperative comorbidities (diabetes mellitus, coronary artery disease, recent surgery, venous stasis), elevated Platelet (PLT), D-dimer, Ratio of neutrophils to lymphocytes (NLR), Erythrocyte sedimentation rate (ESR), C-reactive protein (CPR), IL-6 (Interleukin-6), Procalcitonin (PCT), reduced red blood cell, advanced age, and AB blood group as high-risk factors for preoperative DVT in TKA patients.

The nomogram, serving as a predictive tool for assessing disease risk and prognostic evaluation, has garnered increasing attention and application within medical research and clinical practice. This graphical tool transforms intricate regression equations into visual graphs, facilitating clinicians in computing disease occurrence probabilities and assessing patient prognoses through these graphs [16, 17].

Although the prevention of DVT after TKA has received sufficient attention from clinicians, the importance of preoperative DVT in this process has been overlooked. Existing studies have only reported the incidence and risk factors of preoperative DVT in TKA, but the incidence and risk factors are not consistent. To our knowledge, no study has constructed a prediction model for the occurrence of preoperative DVT in TKA. Therefore, in this study, we analyzed the independent risk factors contributing to preoperative DVT in TKA and constructed a prediction model to accurately evaluate its occurrence based on these factors. By assessing individual risk, clinicians are able to implement additional life monitoring and necessary medical interventions to effectively prevent the progression of DVT.

## Methods

### Patients

We established and validated a nomogram to predict preoperative lower extremity deep vein thrombosis in patients undergoing total knee arthroplasty. All data were obtained from the patient data database of the Second Hospital of Shanxi Medical University.

**Fig. 1:**
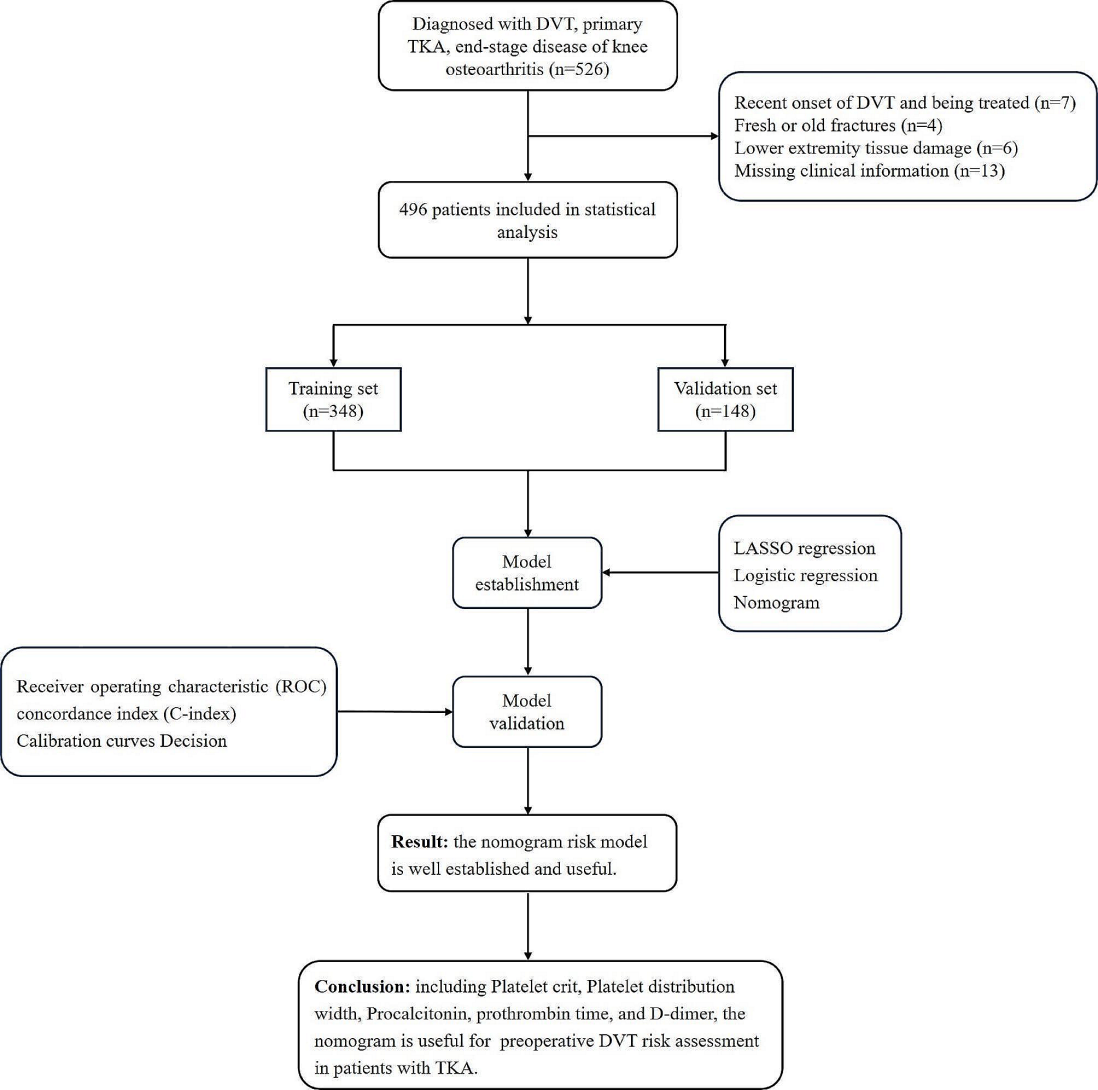
Flow diagram of study design

In the research, data was acquired from 526 patients who underwent TKA at the Second Hospital of Shanxi Medical University from June 2022 to June 2023. Among them, 28 patients were excluded due to recent DVT undergoing treatment, lower extremity fracture, lower extremity tissue injury, and loss of clinical information, leaving a total of 496 patients for the final analysis (Fig. 1). These 496 patients were retrospectively divided into DVT group (*n* = 28) and non-DVT group (*n* = 468) according to the presence of DVT before surgery. Inclusion criteria: (1) patients who underwent elective total knee arthroplasty; (2) patients who underwent preoperative thrombus screening of bilateral lower extremity veins using color Doppler ultrasonography of the lower extremity veins; (3) patients with complete personal information and medical consultation records. Exclusion criteria: (1) patients who were informed of the existence of VTE when they were admitted to the hospital; (2) patients who had recently suffered from venous thromboembolism and were undergoing treatment; (3) patients with fresh or old lower extremity fractures, and patients who needed initial or revision replacement surgery for lower extremity fractures; (4) patients with lower extremity tissue damage that could not be performed by lower extremity venous ultrasonography; (5) patients who had incomplete personal information and medical records. The study was approved by the Ethics Committee of Second Hospital of Shanxi Medical University (approval No. 2023KYNO. 307).

### Procedure

The complete dataset was randomly partitioned into a training set (*n* = 348) and a validation set (*n* = 148) in a ratio of 7:3. All patients underwent pulsed Doppler ultrasonography of both lower extremities 1–3 days before surgery, a procedure performed by an experienced sonographer. The clinical data of the patients were accessed through an electronic medical record system. Clinical information included basic characteristics, chronic comorbidities, and laboratory and ancillary tests. Basic characteristics included name, hospitalization number, height, weight, gender, age and calculated body mass index (BMI). Chronic comorbidities included hypertension, diabetes mellitus, coronary artery disease, history of smoking and alcohol consumption, and history of previous surgery. Laboratory tests and ancillary tests included white blood cell, red blood cell, hemoglobin, hematocrit, mean corpuscular volume, mean corpuscular hemoglobin, mean corpuscular hemoglobin concentration, standard deviation in red blood cell volume distribution width (RDW-SD), coefficient variation of red blood cell volume distribution width (RDW-CV), platelet, platelet crit (PCT), mean platelet volume, platelet-larger cell ratio, platelet distribution width (PDW), neutrophil (NEU), lymphocyte, monocyte, eosinophils, basophil, neutrophil%, lymphocyte%, monocyte%, eosinophils%, basophil%, alanine amino transferase (ALT), aspartate amino transferase (AST), AST/ALT, total bilirubin, direct bilirubin, indirect bilirubin, total protein, serum albumin, globulin, albumin-globulin ratio, blood urea nitrogen, creatinine, blood potassium, blood sodium, blood chloride, blood calcium, procalcitonin (PCT), blood glucose, C-reactive protein, prothrombin time (PT), international normalized ratio, plasminogen activity, fibrinogen, activated partial thromboplastin time (APTT), activated partial thromboplastin time ratio, thrombin time (TT), and D-dimer. If subjects underwent multiple hematological investigations prior to the diagnosis of DVT, we chose to include the most recent one.

### Statistical analysis

The data were categorized into continuous and categorical variables. Normally distributed continuous variables were expressed as mean ± standard deviation, non-normally distributed continuous variables were expressed as median, and categorical variables were expressed as counts (%). Statistical analysis using R software (Version 4.3.1; https://www.r-project.org/). Comparisons between groups of continuous variables were made using t-tests. Comparisons between groups for categorical variables were made using the chi-square test. To identify the most predictive variables for DVT development in TKA patients, the least absolute shrinkage and selection operator (LASSO) was initially employed, suitable for managing high-dimensional data [18, 19]. It is a linear regression method of variable selection and model compression that is able to shrunk the regression coefficients of the variables to zero by the penalty coefficients of lambda. It excluded variables with zero regression coefficients and selected variables without zero regression coefficients. The selected variables were considered to be the most relevant to the occurrence of DVT. The selected variables were subsequently subjected to multifactorial logistic regression analysis, and the variables were evaluated by odds ratios (ORs) and 95% confidence intervals (95%CI) [20]. A two-sided significance level was applied. Further, a nomogram regarding the risk of preoperative DVT development in TKA patients was drawn using the screened variables with *p* < 0.05 by “RMS” package in R language software.

The discriminatory power of the nomogram was Subsequently measured by the area under the curve (AUC) of the receiver operating characteristic (ROC), which is able to show the ability of the nomogram to discriminate between different results. In addition, the calibration curve was constructed to evaluate the calibration effectiveness of the nomogram. Simultaneously, the discriminative capacity of the nomogram was evaluated and analyzed through the concordance index (C-index) [21]. In this cohort, the C-index of the relative correction of the nomogram was also calculated (1000 bootstrap resamples) [22]. Finally, the therapeutic effectiveness of the nomogram was assessed using decision curve analysis, which consists of estimating the net benefit at different threshold probabilities in the cohort [23].

## Results

### Characteristics of the patients

A total of 496 patients who underwent TKA were finally enrolled in this study, of which 28 patients were examined for lower extremity DVT preoperatively, and the prevalence of preoperative lower extremity DVT in patients who underwent TKA was 5.65%. The characteristics of the subjects are shown in Table 1. All subjects were randomly divided into a training set (*n* = 348) and a validation set (*n* = 148) at a ratio of 7:3. The differences between the two populations were not statistically significant in terms of comparison of the clinical data (including general data and data from laboratory and auxiliary examinations) (*P* > 0.05, Table 2).

**Table 1:** Characteristics of patients with DVT group and non-DVT group

Characteristics	DVT group	Non-DVT group	*p* value
Age	66.96 ± 7.57	68.14 ± 6.26	0.342
BMI	24.95 ± 2.75	26.08 ± 3.46	0.091
High blood pressure			0.878
Yes	14 (50%)	241 (51.5%)	
No	14 (50%)	227 (48.5%)	
Diabetes mellitus			0.770
Yes	4 (14.3%)	59 (12.6%)	
No	24 (85.7%)	409 (87.4%)	
Coronary heart disease			1
Yes	1 (3.6%)	32 (6.8%)	
No	27 (96.4%)	436 (93.2%)	
White blood cell			0.108
Abnormal	3 (10.7%)	18 (3.8%)	
Normal	25 (89.3%)	450 (96.2%)	
Red blood cell			0.598
Abnormal	11 (39.3%)	161 (34.4%)	
Normal	17 (60.7%)	307 (65.6%)	
Hematocrit			0.182
Abnormal	14 (50%)	175 (37.4%)	
Normal	14 (50%)	293 (62.6%)	
Mean Corpuscular Volume			0.335
Abnormal	2 (7.1%)	19 (4.1%)	
Normal	26 (92.9%)	449 (95.9%)	
Mean corpuscular hemoglobin			0.623
Abnormal	0 (0%)	21 (4.5%)	
Normal	28 (100%)	447 (95.5%)	
Mean corpuscular hemoglobin concentration			1
Abnormal	2 (7.1%)	44 (9.4%)	
Normal	26 (92.9%)	424 (90.6%)	
Red blood cell volume distribution width SD			0.436
Abnormal	7 (25%)	89 (19%)	
Normal	21 (75%)	379 (81%)	
Red blood cell volume distribution width CV			0.606
Abnormal	6 (21.4%)	80 (17.1%)	
Normal	22 (78.6%)	388 (82.9%)	
Platelet			0.220
Abnormal	3 (10.7%)	26 (5.6%)	
Normal	25 (89.3%)	442 (94.4%)	
Platelet crit			0.003
Abnormal	11 (39.3%)	80 (17.1%)	
Normal	17 (60.7%)	388 (82.9%)	
Mean Platelet Volume			0.381
Characteristics	DVT group	Non-DVT group	*p* value
Abnormal	5 (17.9%)	118 (25.2%)	
Normal	23 (82.1%)	350 (74.8%)	
Platelet-larger cell ratio			0.634
Abnormal	8 (28.6%)	115 (24.6%)	
Normal	20 (71.4%)	353 (75.4%)	
Platelet distribution width			0.014
Abnormal	8 (28.6%)	246 (52.6%)	
Normal	20 (71.4%)	222 (47.4%)	
Neutrophil			0.029
Abnormal	4 (14.3%)	18 (3.8%)	
Normal	24 (85.7%)	450 (96.2%)	
Lymphocyte			1
Abnormal	3 (10.7%)	50 (10.7%)	
Normal	25 (89.3%)	418 (89.3%)	
Monocyte			1
Abnormal	3 (10.7%)	56 (12%)	
Normal	25 (89.3%)	412 (88.0%)	
Eosinophils			0.659
Abnormal	2 (7.4%)	25 (5.3%)	
Normal	26 (92.9%)	443 (94.7%)	
Basophil			0.214
≥ 0.06	5 (17.9%)	49 (10.5%)	
<0.06	23 (82.1%)	419 (89.5%)	
Neutrophil%			0.161
Abnormal	3 (10.7%)	22 (4.7%)	
Normal	25 (89.3%)	446 (95.3%)	
Lymphocyte%			0.102
Abnormal	6 (21.4%)	46 (9.8%)	
Normal	22 (78.6%)	422 (90.2%)	
Monocyte%			0.071
Abnormal	5 (17.9%)	36 (7.7%)	
Normal	23 (82.1%)	432 (92.3%)	
Eosinophils%			0.709
Abnormal	2 (7.1%)	31 (6.6%)	
Normal	26 (92.9%)	437 (93.4%)	
Basophil%			0.283
≥ 1	4 (14.3%)	38 (8.1%)	
<1	24 (85.7%)	430 (91.9%)	
Alanine amino transferase			0.054
Abnormal	5 (17.9%)	33 (7.1%)	
Normal	23 (82.1%)	435 (92.9%)	
Aspartate amino transferase			0.008
Abnormal	7 (25%)	37 (7.9%)	
Characteristics	DVT group	Non-DVT group	*p* value
Normal	21 (75%)	431 (92.1%)	
AST/ALT			1
Abnormal	0 (0%)	8 (1.7%)	
Normal	28 (100%)	460 (98.3%)	
Total bilirubin			0.619
>21	0 (0%)	20 (4.3%)	
≤ 21	28 (100%)	448 (95.7%)	
Direct bilirubin			0.292
>4	2 (10.5%)	17 (89.5%)	
≤ 4	26 (92.9%)	451 (96.4%)	
Indirect bilirubin			0.387
Abnormal	0 (0%)	26 (5.6%)	
Normal	28 (100%)	442 (94.4%)	
Total protein			0.030
Abnormal	9 (32.1%)	71 (15.2%)	
Normal	19 (67.9%)	397 (84.8%)	
Serum albumin			0.216
Abnormal	15 (53.6%)	195 (41.7%)	
Normal	13 (46.4%)	273 (58.3%)	
A/G			0.964
Abnormal	6 (21.4%)	102 (21.8%)	
Normal	22 (78.6%)	366 (78.2%)	
Blood Urea Nitrogen			1
Abnormal	4 (14.3%)	80 (17.1%)	
Normal	24 (85.7%)	388 (82.9%)	
Creatinine			0.177
Abnormal	3 (10.7%)	100 (21.4%)	
Normal	25 (89.3%)	368 (78.6%)	
K			1
Abnormal	3 (10.7%)	52 (11.1%)	
Normal	25 (89.3%)	416 (88.9%)	
Na			1
Abnormal	2 (7.1%)	32 (6.8%)	
Normal	26 (92.9%)	436 (93.2%)	
CL			0.283
Abnormal	4 (14.3%)	38 (8.1%)	
Normal	24 (85.7%)	430 (91.9%)	
Ca			0.475
Abnormal	3 (10.7%)	36 (7.7%)	
Normal	25 (89.3%)	432 (92.3%)	
Procalcitonin			0.000
≥ 0.51	12 (42.9%)	24 (5.1%)	
<0.51	16 (57.1%)	444 (94.9%)	
Characteristics	DVT group	Non-DVT group	*p* value
PT			0.000
Abnormal	15 (53.6%)	55 (11.8%)	
Normal	13 (46.4%)	413 (88.2%)	
INR			1
Abnormal	0 (0%)	4 (0.9%)	
Normal	28 (100%)	464 (99.1%)	
PT%			1
Abnormal	1 (3.8%)	18 (3.8%)	
Normal	27 (96.4%)	450 (96.2%)	
FIB			1
Abnormal	2 (7.1%)	43 (9.2%)	
Normal	26 (92.9%)	425 (90.8%)	
APTT			0.007
Abnormal	4 (14.3%)	11 (2.4%)	
Normal	24 (85.7%)	457 (97.6%)	
TT			1
Abnormal	0 (0%)	11 (2.4%)	
Normal	28 (100%)	457 (97.6%)	
D-Dimer			0.000
≥ 0.55	18 (64.3%)	121 (25.9%)	
<0.55	10 (35.7%)	347 (74.1%)	
C reactive protein			0.574
≥ 5	5 (17.9%)	65 (13.9%)	
<5	23 (82.1%)	403 (86.1%)	

**Abbreviations**: *BMI*: Body mass index, *ALT*: Alanine amino transferase, *AST*: Aspartate amino transferase, *PT*: Prothrombin time, *INR*: International normalized ratio, *FIB*: Fibrinogen, *APTT*: Activated partial thromboplastin time, *TT*: Thrombin time

**Table 2:** Characteristics of training and validation sets

Characteristics	Training set	Validation set	*p* value
Age	67.89 ± 6.35	68.50 ± 6.32	0.292
BMI	26.16 ± 3.55	25.68 ± 3.10	0.369
Gender			0.368
Male	122 (35.1%)	45 (30.4%)	
Female	226 (64.9%)	103 (69.6%)	
High blood pressure			0.503
Yes	175 (50.3%)	80 (54.1%)	
No	173 (49.7%)	68 (45.9%)	
Diabetes mellitus			0.702
Yes	46 (13.2%)	17 (11.5%)	
No	302 (86.8%)	131 (88.5%)	
Coronary heart disease			0.797
Yes	22 (6.3%)	11 (7.4%)	
Characteristics	Training set	Validation set	*p* value
No	326 (93.7%)	137 (92.6%)	
White blood cell			1
Abnormal	15 (4.3%)	6 (4.1%)	
Normal	333 (95.7%)	142 (95.9%)	
Red blood cell			0.653
Abnormal	118 (33.9%)	54 (36.5%)	
Normal	230 (66.1%)	94 (63.5%)	
Hemoglobin			1
Abnormal	0 (0%)	0 (0%)	
Normal	348 (100%)	148 (100%)	
Hematocrit			0.671
Abnormal	130 (37.4%)	59 (39.9%)	
Normal	218 (62.6%)	89 (60.1%)	
Mean Corpuscular Volume			0.115
Abnormal	11 (3.2%)	10 (6.8%)	
Normal	337 (96.8%)	138 (93.2%)	
Mean corpuscular hemoglobin			0.548
Abnormal	13 (3.7%)	8 (5.4%)	
Normal	335 (96.3%)	140 (94.6%)	
Mean corpuscular hemoglobin concentration			0.451
Abnormal	35 (10.1%)	11 (7.4%)	
Normal	313 (89.9%)	137 (92.6%)	
Red blood cell volume distribution width SD			0.303
Abnormal	72 (20.7%)	24 (16.2%)	
Normal	276 (79.3%)	124 (83.8%)	
Red blood cell volume distribution width CV			0.828
Abnormal	59 (17%)	27 (18.2%)	
Normal	289 (83%)	121(81.8%)	
Platelet			0.723
Abnormal	19 (5.5%)	10 (6.8%)	
Normal	329 (94.5%)	138 (93.2%)	
Platelet crit			0.552
Abnormal	61 (17.5%)	30 (20.3%)	
Normal	287 (82.5%)	118 (79.7%)	
Mean Platelet Volume			0.963
Abnormal	87 (25%)	36 (24.3%)	
Normal	261 (75%)	112 (75.7%)	
Platelet-larger cell ratio			0.963
Abnormal	87 (25%)	36 (24.3%)	
Normal	261 (75%)	112 (75.7%)	
Platelet distribution width			0.262
Abnormal	172 (49.4%)	82 (55.4%)	
Normal	176 (50.6%)	66 (44.6%)	
Characteristics	Training set	Validation set	*p* value
Neutrophil			0.325
Abnormal	18 (5.2%)	4 (2.7%)	
Normal	330 (94.8%)	144 (97.3%)	
Lymphocyte			0.394
Abnormal	34 (9.8%)	19 (12.8%)	
Normal	314 (90.2%)	129 (87.2%)	
Monocyte			0.074
Abnormal	35 (10.1%)	24 (16.2%)	
Normal	313 (89.9%)	124 (83.8%)	
Eosinophils			1
Abnormal	329 (94.5%)	140 (94.6%)	
Normal	19 (5.5%)	8 (5.4%)	
Basophil			0.410
≥ 0.06	41 (11.8%)	13 (8.8%)	
<0.06	307 (88.2%)	135 (91.2%)	
Neutrophil%			0.641
Abnormal	16 (4.6%)	9 (6.1%)	
Normal	332 (95.4%)	139 (93.9%)	
Lymphocyte%			0.202
Abnormal	32 (9.2%)	20 (13.5%)	
Normal	316 (90.8%)	128 (86.5%)	
Monocyte%			0.330
Abnormal	316 (90.8%)	139 (93.9%)	
Normal	32 (9.2%)	9 (6.1%)	
Eosinophils%			1
Abnormal	23 (6.6%)	10 (6.8%)	
Normal	325 (93.4%)	138 (93.2%)	
Basophil%			0.285
≥ 1	33 (9.5%)	9 (6.1%)	
<1	315 (90.5%)	139 (93.9%)	
Alanine amino transferase			0.953
Abnormal	26 (7.5%)	12 (8.1%)	
Normal	322 (92.5%)	136 (91.9%)	
Aspartate amino transferase			0.828
Abnormal	32 (9.2%)	12 (8.1%)	
Normal	316 (90.8%)	136 (91.9%)	
AST/ALT			0.247
Abnormal	4 (1.1%)	4 (2.7%)	
Normal	344 (98.9%)	144 (97.3%)	
Total bilirubin			1
>21	14 (4%)	6 (4.1%)	
≤ 21	334 (96%)	142 (95.9%)	
Direct bilirubin			1
Characteristics	Training set	Validation set	*p* value
>4	13 (3.7%)	6 (4.1%)	
≤ 4	335 (96.3%)	142 (95.9%)	
Indirect bilirubin			1
Abnormal	330 (94.8%)	140 (94.6%)	
Normal	18 (5.2%)	8 (5.4%)	
Total protein			0.368
Abnormal	288 (82.8%)	128 (86.5%)	
Normal	60 (17.2%)	20 (13.5%)	
Serum albumin			0.409
Abnormal	152 (43.7%)	58 (39.2%)	
Normal	196 (56.3%)	90 (60.8%)	
Globulin			1
Abnormal	0 (0%)	0 (0%)	
Normal	348 (100%)	148 (100%)	
A/G			0.517
Abnormal	79 (22.7%)	29 (19.6%)	
Normal	269 (77.3%)	119 (80.4%)	
Blood Urea Nitrogen			0.502
Abnormal	62 (17.8%)	22 (14.9%)	
Normal	286 (82.2%)	126 (85.1%)	
Creatinine			0.955
Abnormal	73 (21%)	30 (20.3%)	
Normal	275 (79%)	118 (79.7%)	
K			0.201
Abnormal	34 (9.8%)	21 (14.2%)	
Normal	314 (90.2%)	127 (85.8%)	
Na			0.193
Abnormal	20 (5.7%)	14 (9.5%)	
Normal	328 (94.3%)	134 (90.5%)	
CL			1
Abnormal	29 (8.3%)	13 (8.8%)	
Normal	319 (91.7%)	135 (91.2%)	
Ca			1
Abnormal	27 (7.8%)	12 (8.1%)	
Normal	321 (92.2%)	136 (91.9%)	
Procalcitonin			0.639
≥ 0.51	27 (7.8%)	9 (6.1%)	
<0.51	321 (92.2%)	139 (93.9%)	
Glucose			
Abnormal	0 (0%)	0 (0%)	
Normal	348 (100%)	148 (100%)	
PT			0.194
Abnormal	44 (12.6%)	26 (17.6%)	
Characteristics	Training set	Validation set	*p* value
Normal	304 (87.4%)	122 (82.4%)	
INR			0.323
Abnormal	4 (1.1%)	0 (0%)	
Normal	344 (98.9%)	148 (100%)	
PT%			0.267
Abnormal	16 (4.6%)	3 (2%)	
Normal	332 (95.4%)	145 (98%)	
FIB			0.714
Abnormal	30 (8.6%)	15 (10.1%)	
Normal	318 (91.4%)	133 (89.9%)	
APTT			0.250
Abnormal	13 (3.7%)	2 (1.4%)	
Normal	335 (96.3%)	146 (98.6%)	
TT			0.740
Abnormal	7 (2%)	4 (2.7%)	
Normal	341 (98%)	144 (97.3%)	
D-Dimer			0.658
≥ 0.55	95 (27.3%)	44 (29.7%)	
<0.55	253 (72.7%)	104 (70.3%)	
C reactive protein			0.863
<5	48 (13.8%)	22 (14.9%)	
≥ 5	300 (86.2%)	126 (85.1%)	

**Abbreviations**: *BMI*: Body mass index, *ALT*: Alanine amino transferase, *AST*: Aspartate amino transferase, *ALB*: Serum albumin, *GLO*: Globulin, *PT*: Prothrombin time, *INR*: International normalized ratio, *FIB*: Fibrinogen, *APTT*: Activated partial thromboplastin time, *TT*: Thrombin time

### Lasso regression and multifactor logistic regression analysis

**Fig. 2:**
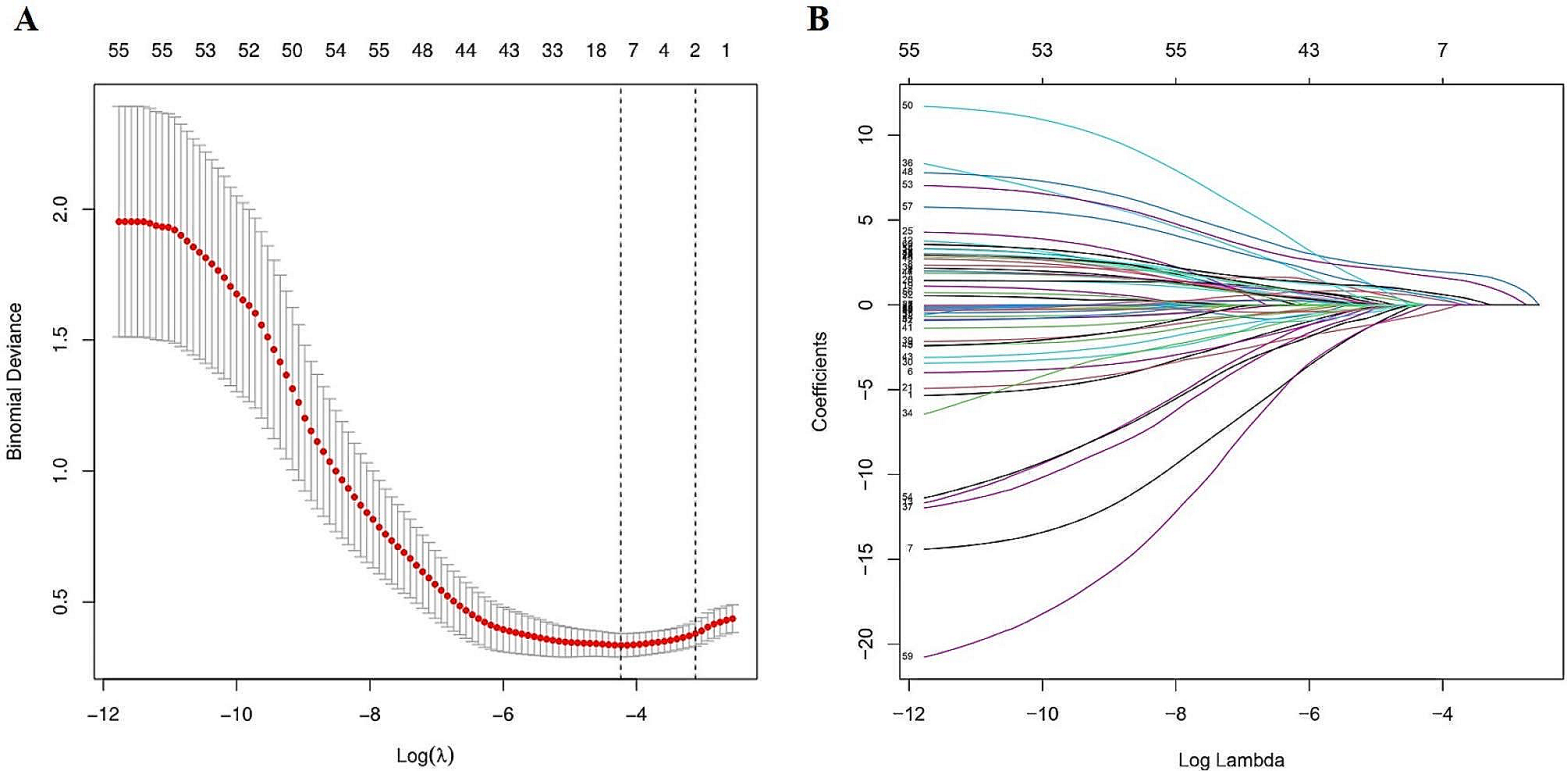
Demographic and clinical risk factors selection using the LASSO binary logistic regression model. (**A**) The lasso model was cross-validated using the minimum criterion, with dashed vertical lines plotted at the optimal values (8 factors). (**B**) The 55 feature LASSO coefficient profiles for logarithmic (lambda) sequences are constructed

First, initial screening for predictors (nonzero coefficients) of preoperative occurrence of lower extremity DVT in TKA was performed by Lasso regression analysis in the modeled population. Variables were pooled and normalized by 10-fold cross-validation (Fig. 2). The selected predictors were Platelet crit, Platelet distribution width, Neutrophil, Aspartate amino transferase, Procalcitonin, prothrombin time, activated partial thromboplastin time and D-dimer. Subsequent multifactorial logistic regression analysis indicated that Platelet crit, Platelet distribution width, Procalcitonin, prothrombin time, and D-dimer were independent risk factors for preoperative development of lower extremity DVT in patients undergoing TKA (Table 3).

**Table 3:** Prediction factors for the risk of preoperative DVT development nomogram

Intercept and variable	Prediction model		
	β	Odds ratio (95% CI)	*P*-value
Intercept	-5.047	0.006 (0.001–0.022)	<0.001
Platelet crit	2.217	9.182 (2.262–41.210)	0.002
Platelet distribution width	-1.630	0.196 (0.041–0.752)	0.026
Neutrophil	0.454	1.574 (0.251–8.119)	0.603
Aspartate amino transferase	0.862	2.369 (0.389–12.933)	0.328
Procalcitonin	3.094	22.074 (5.214-110.884)	<0.001
PT	2.378	10.778 (2.866–45.216)	<0.001
APTT	1.660	5.262 (0.811–35.474)	0.079
D-dimer	1.487	4.424 (1.347–15.612)	0.016

**Note**: *β*is the regression coefficient

**Abbreviations**: *CI*: Confidence interval, *PT*: Prothrombin time, *APTT*: Activated partial thromboplastin time

### Modeling of nomogram prediction

**Fig. 3:**
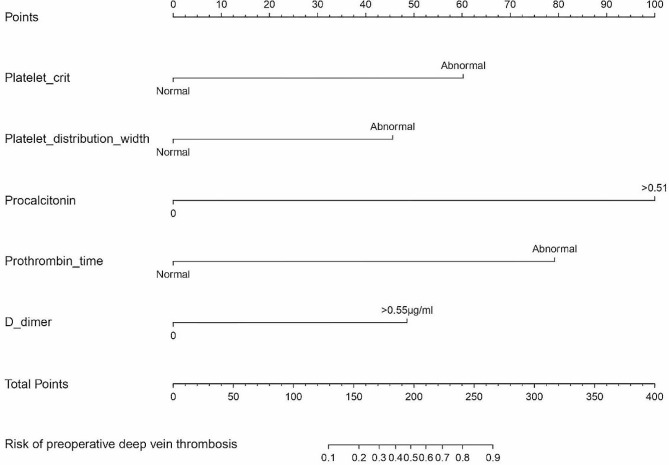
Development of a nomogram for predicting the risk of preoperative DVT development in TKA patients

The five independent risk factors mentioned above were included and successfully constructed a nomogram for the preoperative occurrence of lower extremity DVT in patients undergoing TKA (Fig. 3). This nomogram assigns individual scores based on the five independent factors, and their sum culminates in a total score. The predicted probability corresponding to the total score is the risk of preoperative occurrence of lower extremity DVT in patients undergoing TKA.

### Evaluation and validation of nomogram

**Fig. 4:**
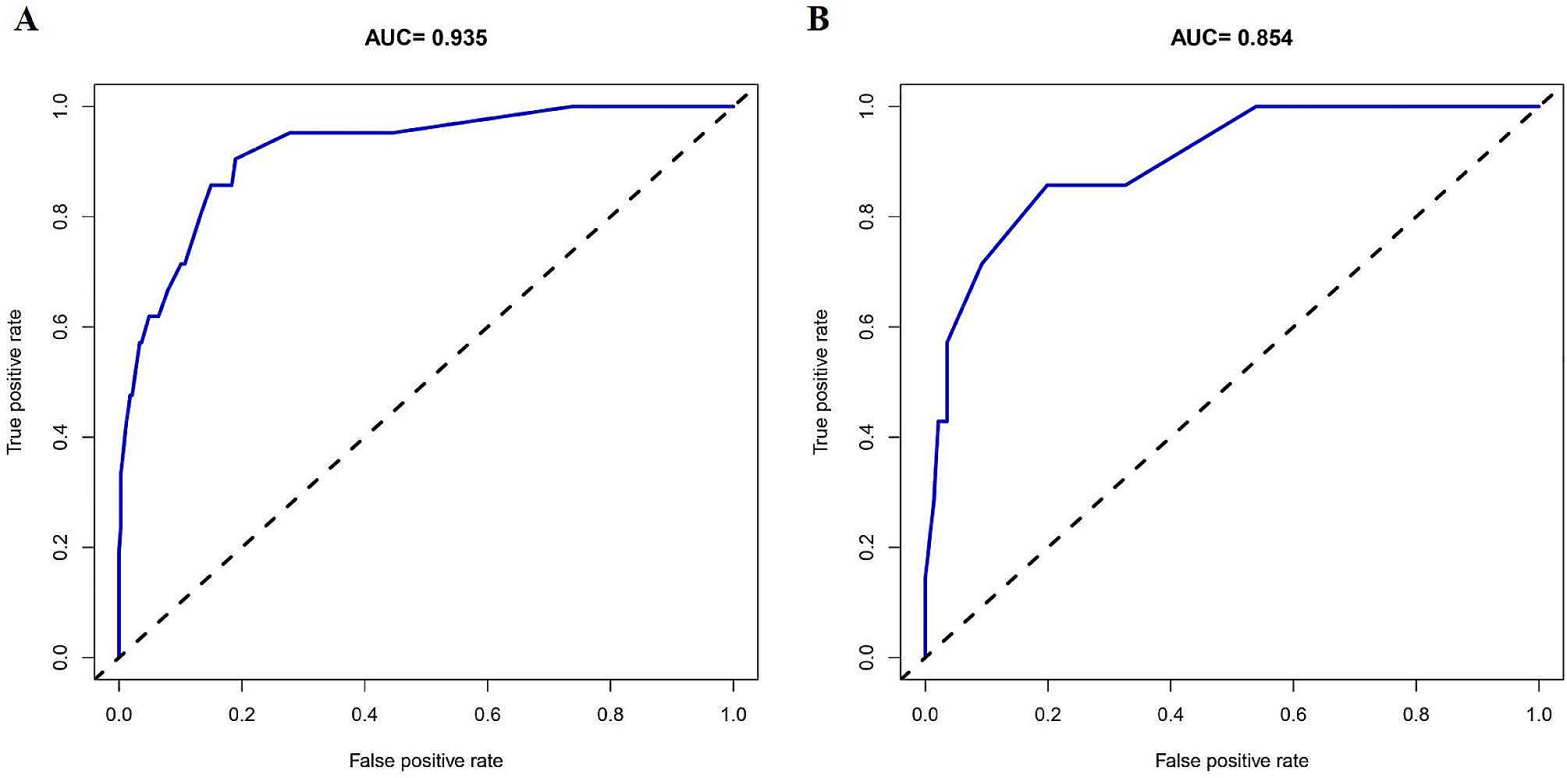
Receiver-operating characteristic (ROC) analysis of the risk of preoperative DVT development nomograms in the training (**A**) and validation (**B**) sets

By plotting the ROC curves of the two populations, AUCs of 0.935 (95%CI: 0.880–0.990) and 0.854 (95%CI: 0.697-1.000) were obtained, indicating the great discriminative capability of the predictive model (Fig. 4).

**Fig. 5:**
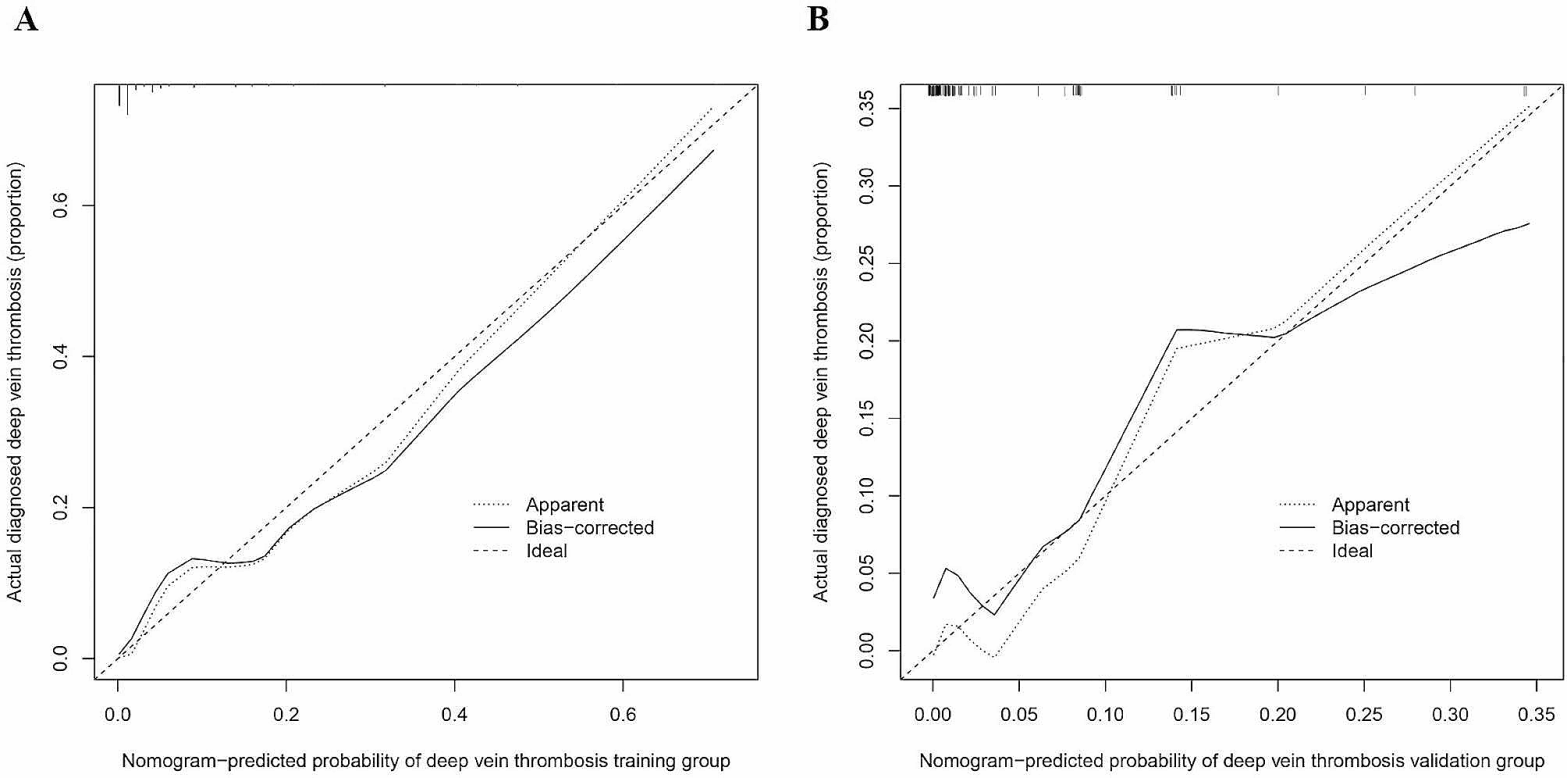
Calibration curve for the risk of preoperative DVT development nomogram in the training (**A**) and validation (**B**) sets. The X-axis indicates the predicted probability of the model and the Y-axis indicates the actual probability. The diagonal dashed line represents a perfect prediction of the ideal model. The solid line presents the performance of the nomogram, and the closer the two lines fit to the diagonal dashed line, the better the predictive consistency of the nomogram

**Fig. 6:**
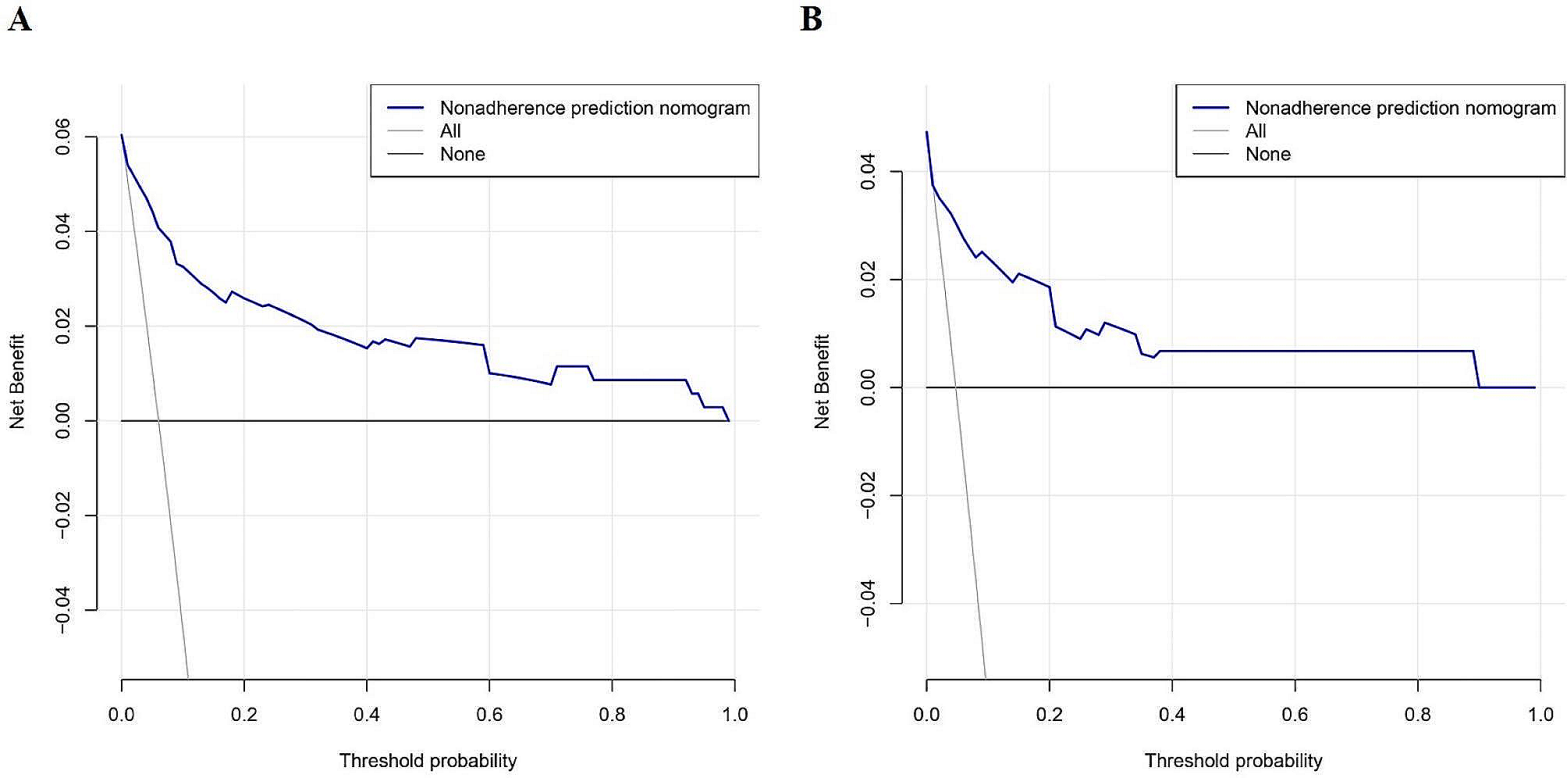
Decision curve analysis for the risk of preoperative DVT development nomogram in the training (**A**) and validation (**B**) sets. DCA illustrated that the net benefit of the training model is higher in the threshold probability interval of 1–98%, and the net benefit of the validation model is higher in the threshold probability interval of 1–90%

Calibration curves showed that the mean absolute error (MAE) was 0.029 and 0.013 for the two populations, indicating that the predictive model was highly calibrated (Fig. 5). The C-indexes of the two populations were 0.919 (95% CI: 0.860–0.978) and 0.900 (95% CI: 0.791–1.009), indicating the favorable discriminative capacity of the predictive model (Fig. 5). When the threshold probability values of the decision curves for the two populations were in the range of 1-98% and 1-90%, the net benefit of using the nomogram to predict the risk of developing lower extremity DVT was better, denoting the strong clinical utility of the predictive model (Fig. 6).

## Discussion

Lower extremity deep vein thrombosis is a common complication of knee arthroplasty, which brings a heavy burden to patients and society [24]. In recent years, color Doppler ultrasound has been widely used in the clinical diagnosis of thrombosis because of its simplicity and reliability [25], however, there is a lag in its results. Therefore, identifying DVT risk factors can enable clinicians to timely adjust treatment measures based on patient conditions.

Nomogram can translate intricate data into predictive clinical models through mathematical modeling [16, 17]. And no study has developed a prediction model for preoperative DVT occurrence in TKA. Therefore, we compiled and analyzed the clinical data of patients who underwent TKA at our center over the past year, and constructed a user-friendly clinical prediction model based on their clinical characteristics and risk factors associated with the formation of preoperative DVT, in order to assess the risk and take appropriate interventions.

Based on previously published results, five influential factors most associated with the occurrence of preoperative DVT in TKA were screened by lasso regression and multifactorial logistic regression analyses and a nomogram was constructed. These factors included Platelet crit, Platelet distribution width, Procalcitonin, prothrombin time, and D-dimer. These predictors incorporated in the model were common and readily available. The model exhibited great predictive ability in both the training and validation sets, demonstrating robust clinical utility in foreseeing preoperative lower extremity DVT events in patients undergoing TKA.

The incidence of preoperative DVT in patients who underwent TKA in this study was 5.6%, which is similar to the results reported by Kim et al. Kim KI et al. screened 311 patients with osteoarthritis for DVT before knee arthroplasty, reporting a 4.5% preoperative DVT incidence in TKA [26]. The incidence of preoperative thrombosis has also been reported in the literature to be higher than in this study. Watanabe H et al. used 16-row multidetector computed tomography to screen for thrombosis in 71 patients undergoing knee arthroplasty both preoperatively and postoperatively, and found a preoperative thromboembolism rate of 8% [27]. Additionally, Wakabayashi H et al. used ultrasound to screen for DVT before surgery in 322 patients undergoing knee arthroplasty, revealing a notably higher incidence of 17.4% preoperative DVT [28]. The reasons for the different incidence of preoperative DVT in TKA may be related to the demographic characteristics of the study population and differences in medical history.

A number of studies have screened the risk factors for preoperative DVT formation in TKA patients, and then investigated the predictive value of individual factors for DVT formation. Xiong X et al. collected and statistically analyzed the clinical data of 458 patients who underwent TKA, discovering that several serological indices, including Platelet crit, Platelet distribution width, Procalcitonin, and D-dimer, were independent risk factors for the development of preoperative DVT in TKA patients [15]. Platelet crit, a parameter evaluating platelet count and concentration, emerges as a biomarker linked to DVT development prior to TKA [29]. Another study by Xiong X et al. concluded that a PCT > 0.228% is an independent risk factor for the development of DVT before TKA [30]. PCT plays a crucial role in regulating normal hemostasis and coagulation processes, and abnormal levels indicate potential platelet count and function irregularities, thereby elevating thrombosis risk. Platelet distribution width reflects the degree of platelet variability and is a marker of platelet activation [31]. Öztürk ZA et al. observed significantly lower PDW levels in the active phase of ulcerative colitis and Crohn’s disease compared to the remission phase, suggesting that a decrease in the PDW may be related to progression or activation of the disease rather than the disease itself [32]. Ma J et al. found that a decrease in the PDW was significantly associated with the occurrence of DVT [33]. A decrease in PDW signifies heightened platelet homogeneity and increased platelet activity, potentially contributing to DVT development during hypercoagulable states in the blood. D-dimer is derived from cross-linked fibrin clots dissolved by fibrinolytic enzymes, serving as a sensitive biomarker indicative of fibrinolytic activity and coagulation function [34]. Therefore, it holds significance in thrombus screening. Stamou KM et al. noted that persistently high levels of D-dimer in the early stages of trauma not only reflect fibrinolytic activity and coagulation, but also hints at the formation of inconspicuous microthrombi [35]. However, D-dimer can be affected by a variety of factors in the body, such as trauma, infection, and tumor [36]. Our study, which excluded these confounding factors, confirm that D-dimer may be an important risk factor for preoperative lower extremity deep vein thrombosis in patients undergoing TKA. This is consistent with the findings of Jiang et al. who found that D-dimer > 0.5 µg/ml in end-stage osteoarthritis was a risk factor for DVT in patients hospitalized for TKA [37]. The mechanism behind this association may involve activated fibronectin during thrombosis, leading to increased D-dimer expression. The Prothrombin Time serves as a vital marker in coagulation screening assays, detecting the normalization of exogenous coagulation pathways and common bodily pathways [38]. Nevertheless, no study has identified the potential value of PT in predicting the risk of preoperative DVT in patients with TKA. Cao et al. compared clinical data between people who developed DVT after fracture with those who did not develop DVT after fracture and with healthy controls. They observed that the level of PT was significantly increased in the DVT group, and they noted that the optimal threshold for PT to diagnose DVT was 12.05s, with a sensitivity of 72.92% and specificity of 47.92% (AUC = 0.617, 95% CI 0.505–0.730, *p* = 0.048) [39]. Above all, the predictors included in our prediction model in this study were all inflammatory in previous studies, proving the validity of our study.

In conclusion, the nomogram constructed in this study has high accuracy and may play an important value in the early identification and risk prediction of preoperative occurrence of DVT in patients undergoing TKA. It provides clinicians with more favorable clinical guidance in terms of medical measure interventions. However, our study has some limitations: first, it is a single-center study with a limited sample size, which may limit its generalization and weaken the statistical analysis, thus biasing the results. Secondly, this is a retrospective study with incomplete information on some cases, and incomplete information such as lipids and thrombosis elastogram were discarded in our study, so it may not have included all potential factors affecting the occurrence of DVT. Additionally, our constructed prediction model underwent internal validation exclusively, lacking external validation across multiple centers. Thus, the applicability of our findings to broader populations undergoing TKA in various regions and countries remains uncertain, and external validation in a wider population receiving TKA is needed in subsequent studies to draw more comprehensive and reliable conclusions.

## Conclusion

In this study, a nomogram was successfully constructed to predict the risk of preoperative DVT in patients undergoing TKA. The model demonstrates great discriminatory power, calibration and clinical validity. By assessing individual risk, clinicians can recognize the occurrence of DVT early and thus implement additional life monitoring and necessary medical interventions to effectively prevent the progression of DVT.

## Data Availability

The datasets generated and/or analyzed during the current study are not publicly available due to limitations of ethical approval involving the patient data and anonymity but are available from the corresponding author on reasonable request.
